# Liposomal encapsulation enhances the antitumour efficacy of the vascular disrupting agent ZD6126 in murine B16.F10 melanoma

**DOI:** 10.1038/sj.bjc.6604675

**Published:** 2008-09-16

**Authors:** M H A M Fens, K J Hill, J Issa, S E Ashton, F R Westwood, D C Blakey, G Storm, A J Ryan, R M Schiffelers

**Affiliations:** 1 Department of Pharmaceutics, Utrecht Institute for Pharmaceutical Sciences (UIPS), Utrecht University, Utrecht, The Netherlands; 2 Pharmaceutical and Analytical R&D, AstraZeneca, Mereside, Alderley Park, Macclesfield, Cheshire, UK; 3 Cancer Bioscience, AstraZeneca, Mereside, Alderley Park, Macclesfield, Cheshire, UK; 4 Safety Assessment, AstraZeneca, Mereside, Alderley Park, Macclesfield, Cheshire, UK

**Keywords:** vascular disrupting agents, liposomes, drug targeting, endothelium

## Abstract

Vascular disrupting agents (VDAs) are able to affect selectively tumour endothelial cell morphology resulting in vessel occlusion and widespread tumour cell necrosis. However, single-agent antitumour activity of VDAs is typically limited, as tumour regrowth occurs rapidly following drug treatment. To improve the therapeutic effectiveness of VDAs, we investigated liposomal targeting using ZD6126 as a model VDA. ZD6126 is a phosphate-prodrug of the tubulin-binding vascular disrupting agent ZD6126 phenol. ZD6126 was encapsulated into long circulating PEG-liposomes for passive targeting and PEG-liposomes conjugated with peptide ligands containing the RGD-motif for active targeting to *α*_v_-integrins on tumour endothelial cells. ZD6126 could be stably encapsulated, and liposomes displayed minimal leakage *in vitro* (<10% in 3 weeks). *In vivo*, upon intravenous injection, free ZD6126 was rapidly converted into ZD6126 phenol, which was cleared from the circulation within minutes. In contrast, ZD6126 encapsulated into either RGD-targeted or PEG liposomes showed prolonged blood circulation times (*t*_1/2_=10 h), and ZD6126 phenol exposure was also prolonged (*t*_1/2_=8 h). Both liposomal formulations displayed tumour accumulation plus hepatosplenic uptake by local macrophages. The altered pharmacokinetics and tissue distribution profiles of both liposomal ZD6126 formulations resulted both in single-dose and multiple-dose regimes, in improved therapeutic efficacy in established murine B16.F10 melanomas compared with free ZD6126. The passively and actively targeted liposomes showed equal antitumour efficacy, indicating that delivery of ZD6126 to the tumour tissue may suffice to disrupt tumour blood vessels without the need for specific targeting to the tumour endothelium.

Blood vessel development is important for tumour progression. Therapies that specifically focus on targeting tumour vasculature have been extensively explored since the early 1970s (Folkman, 1971). Vascular-targeted therapies can be divided into two distinct groups: anti-angiogenic agents and vascular disrupting agents. Anti-angiogenic agents prevent new blood vessel growth by interfering with angiogenic stimuli and appear most beneficial in early-stage disease. To prevent tumour growth, these agents need to be dosed chronically over prolonged periods of time to continuously obstruct formation of new tumour blood vessels (Drixler *et al*, 2000). Vascular disrupting agents, on the other hand, have an immediate destructive effect. They disrupt established tumour blood vessels, inducing vascular collapse leading to tumour cell death (Siemann *et al*, 2005; Tozer *et al*, 2005).

Tumour vessels are different from quiescent endothelium at various levels. For example, tumour vasculature generally lacks the structural support of mature vessels, is more permeable, and also expresses distinct antigens (Carmeliet and Jain, 2000). Furthermore, immature angiogenic endothelial cells rely on a microtubule cytoskeleton to support their elongated shape. Microtubule-destabilising vascular disrupting agents (VDAs) act through inhibition of tubulin polymerisation, which results in the activation of RhoA, an intracellular coordinator of cytoskeletal rearrangements, and its downstream effectors (Lippert, 2006). Rearrangement of the cytoskeleton initiates loss of the elongated endothelial cell shape (‘rounding up’) and subsequent basement membrane exposure. Exposed basement membranes may induce thrombus formation causing blood vessel congestion, blood flow reduction, and hypoxia resulting in necrosis and tumour cell death. Certain tubulin binding microtubule-destabilizing agents (including colchicine and vincristine) have only shown VDA effects at doses near the maximum tolerated dose (MTD) (Thorpe *et al*, 2003). In contrast, second-generation tubulin binding microtubule-destabilizing agents display vascular disrupting activity in tumours at doses well below their MTD.

A number of second-generation compounds have been extensively studied in preclinical settings including ZD6126, Combretastatin A4 phosphate, AVE8062, ABT-751, and OXi4503. Pronounced antitumour effects were seen in a wide variety of preclinical tumour models (Blakey *et al*, 2002b; Hill *et al*, 2002a, 2002b). Despite up to 95% necrosis in the tumour mass, tumour progression still occurred within a few days of treatment. To improve their effectiveness, VDAs have been extensively studied in combination with other therapies including radiation (Siemann and Rojiani, 2002b; Wachsberger *et al*, 2005), chemotherapeutic agents (Blakey *et al*, 2002b; Siemann and Rojiani, 2002a; Taraboletti *et al*, 2005), and anti-angiogenic drugs (Siemann and Shi, 2004). Sengupta *et al* (2005) showed improved therapeutic index and reduced toxicity in a co-encapsulated nanoscale formulation of combretastatin A4 and doxorubicin. Because peak blood flow reduction is obtained within a few hours of treatment with a VDA (Goertz *et al*, 2002), and total repopulation of the tumour generally is accomplished within 72 h after treatment (McIntyre *et al*, 2004), there is a limited time frame for combination therapy strategies to be successful, at least in preclinical models.

In clinical trials, this class of compounds has shown marked reductions in tumour blood flow, although single-agent antitumour efficacy has been very limited, as predicted from the preclinical models (Gould *et al*, 2007). We considered that changing the tissue distribution of VDAs by targeting the drug to the tumour vasculature could be a strategy to improve the therapeutic effectiveness of these compounds. One of the most attractive drug delivery systems for tumour targeting are liposomes, as they show a relative high accumulation and have a unparalleled circulatory half life (Allen and Cullis, 2004). Liposomes are spherical nanoparticles containing a phospholipid bilayer surrounding an aqueous core. Addition of a poly(ethylene glycol) (PEG)-coating delays uptake by the mononuclear phagocyte system (MPS), resulting in prolonged circulation time and enhanced tumour accumulation due to the enhanced permeability and retention effect (Vail *et al*, 2004; Minchinton and Tannock, 2006). Furthermore, liposomes can be specifically targeted by coupling peptide ligands or antibodies to the outer surface. Previously, the chemotherapeutic agent doxorubicin was encapsulated into long-circulating liposomes. Liposomal delivery of cytotoxic agent doxorubicin resulted in higher tumour drug levels and enhanced therapeutic efficacy. This is the result of the altered pharmacokinetics and tissue distribution profile of the liposomal formulation (Park, 2002; Safra, 2003; Ewer *et al*, 2004). Doxorubicin-containing liposomes are clinically used for the treatment of several types of cancer (Northfelt *et al*, 1998; Perez *et al*, 2002; Thigpen *et al*, 2005).

In this study, we investigated whether the therapeutic effectiveness of ZD6126 could be increased by targeting the agent to the tumour tissue. ZD6126 is a water-soluble phosphate prodrug of the tubulin-binding agent ZD6126-phenol (*N*-acetylcolchinol), which is formed after rapid hydrolysis of the prodrug by phosphatases present in circulating blood.

Intravenously (i.v.) injected ZD6126 has a very fast elimination rate from the blood in both rats and humans with *t*_1/2_ values of less than 1 h. Altering pharmacokinetics and the tissue distribution profile of ZD6126 by encapsulation into long circulating PEG-liposomes could improve therapeutic efficacy. To further improve site-specific delivery to tumour angiogenic endothelium, we have additionally coupled cyclic RGD (Arg-Gly-Asp) peptides to the surface of the PEG liposomes, which specifically bind to *α*_v_-integrins that are overexpressed on the surface of angiogenic endothelial cells (Thorpe *et al*, 2003).

## Materials and methods

### Preparation of ZD6126-encapsulated liposomes

Long circulating liposomes were prepared as described previously (Schiffelers *et al*, 2005). Composition of the liposomes was dipalmitoylphosphatidylcholine (Lipoid GmbH, Ludwigshafen, Germany), cholesterol (Sigma, St Louis, MO, USA), PEG 2000- distearoylphosphatidylethanolamine (Lipoid GmbH), and in case of ligand targeted liposomes maleimide-PEG 2000, distearoylphosphatidylethanolamine (Lipoid GmbH) was additionally added. Total molar ratio was 1.85 : 1 : 0.15 and 1.85 : 1 : 0.075 : 0.075, respectively, and total lipid amount 100 *μ*M ml^−1^. After dissolving the lipids in chloroform:methanol (2 : 1 vol:vol), a lipid film was made under reduced pressure on a rotary evaporator and dried under a stream of nitrogen. Liposomes were formed by addition of an aqueous solution (40–80 mg ml^−1^) of ZD6126. Approximately 150-nm-sized liposomes (Polydispersity index <0.2) were obtained by repeated extrusion. Encapsulated ZD6126 concentration was determined by high-pressure liquid chromatography (Xterra RP_18_ 3.5 *μ*m, 150 mm long × 4.6 mm ID column) using a gradient of 80% 1000 : 1 water:trifluoroacetic acid (solvent A)/20% 1000 : 1 acetonitrile:trifluoroacetic acid (solvent B) to 10% solvent A and 90% solvent B over 9 min, as a mobile phase.

Cyclic RGD (ARG-GLY-ASP) peptide coupling was performed as described previously (Schiffelers *et al*, 2003). In brief, 4 nM cRGD peptide (JPT, Berlin, Germany) per *μ*M total lipid was added after deacetylation of the peptide in 0.5 M hydroxylamine solution and incubated for 1 h at room temperature. Unloaded ZD6126 and unbound RGD were separated from the liposomes by PD-10 column (Amersham, Piscataway, NJ, USA). To obtain 10 mg ml^−1^ ZD6126 liposomal formulations, liposomes were concentrated by ultracentrifugation for 1 h at 60 000 r.p.m. (Beckman, Fullerton, CA, USA).

### *In vitro* cell toxicity

Human umbilical vein endothelial cells (HUVECs), B16F10 murine melanoma cells and J774A.1 murine tumour-derived macrophages were grown at 37 °C and 5% CO_2_ conditions in EGM-2 (Cambrex, East Rutherford, NJ, USA) and heat-inactivated FBS-supplemented Dulbecco's modified Eagle's medium, respectively. Cells were seeded (1 × 10^4^ cells per well) in 96-well plates 24 h before samples were added. ZD6126 formulations (0.01–100 *μ*M) were incubated with the cells for 24 h and finally an XTT cell viability assay was performed (Scudiero *et al*, 1988).

### Pharmacokinetics studies

PEG-liposomal (RGD-coupled) and free ZD6126 (20 mg kg^−1^) was administered i.v. in the tail vein to C57Bl/6 mice (*n*=3 per group). Blood samples were collected by cardiac puncture at various time intervals (5 min to 48 h) after dosing. Plasma levels of ZD6126 and ZD6126 phenol were determined by tandem mass spectrometry (PE Sciex API 3000 spectrophotometer). The limits of quantification were 10 ng ml^−1^ for ZD6126 and ZD6126 phenol.

### Immunohistochemical analysis

ZD6126-loaded RGD-PEG-liposome (20 mg kg^−1^)-treated B16F10 mice were killed at several time points and tumours (>100 mm^3^), livers and spleens were excised, snap-frozen in liquid nitrogen and stored at −80 °C. After acetone fixation, 5 *μ*m sections were stained for the RGD peptide. Staining of RGD was performed as described previously (Schraa *et al*, 2002). In brief, sections were incubated with polyclonal anti-RGD antibodies raised in rabbits (diluted 1 : 1000 in PBS/FCS 5% buffer), followed by immunoperoxidase staining. Subsequently, a second goat anti-rabbit horseradish peroxidase-labelled antibody (diluted 1 : 100, Dako A/S, Carpinteria, CA, USA) and a third rabbit anti-goat horseradish peroxidase-labelled antibody (1 : 100, Dako A/S) were added. For the F4/80 (pan macrophage marker) staining (Lee *et al*, 1985), monoclonal rat *α*-anti mouse (clone CI:A3-1, Serotec, Raleigh, NC, USA) 1 : 40 (in PBS+BSA 1%) was used as primary antibody after serum blocking with normal rabbit serum (1 : 20 in TBS-Tween 20). Subsequently, staining of endogenous peroxidase and biotin blocking (Vector, Orton, UK) was performed followed by incubation with biotinylated rabbit anti-rat IgG (H+L, 1 : 400 in PBS, Vector) and horseradish peroxidase-streptavidin (1 : 400 in PBS, Vector). For both staining procedures, 3-amino-9-ethylcarbazole (Sigma-Aldrich, St Louis, MO, USA) was used as a substrate and hematoxylin (Sigma-Aldrich) for counterstaining. Slides were mounted in Kaiser's glycerol-gelatin medium (Merck, Darmstadt, Germany) and visualised by light microscopy (Nikon TE2000-U).

### RGD-staining quantification

Microscopic fields (200 × magnified) per sample were analysed for stained regions (sum of substrate-stained pixels) with computerised image analysis programme (Image-Pro® plus 4.5 for windows). Computer software was used to quantify total substrate (red) stained areas. Finally, data were corrected for tissue weight to calculate total staining per gram of tissue.

### Tumour necrosis

Necrosis was assessed by light microscopy. B16.F10 tumour-bearing mice (*n*=10 per group, average tumour size=255±143 mm^3^) were treated with free ZD6126 (100 mg kg^−1^), RGD-liposomal ZD6126 (100 mg kg^−1^) or liposomal PBS control and tumours were excised 24 h afterwards. Subsequent to fixation in 10% buffered formalin and standard processing to paraffin wax blocks, sections (5 *μ*m) were prepared and stained with H&E. The level of necrosis was scored blinded to treatment details by a pathologist. Scores can vary between 1 and 10, where 1 specifies 0–10% necrosis and 10 denotes >90–100% necrosis (Blakey *et al*, 2002b). For the free ZD6126 200 mg kg^−1^ dose, multiple images were fitted together using PhotoFit Harmony software.

### *In vivo* therapeutic efficacy studies

C57Bl/6J mice were injected subcutaneously in the right flank with 1 × 10^6^ B16F10 melanoma cells at day 0. At day 11 after tumour cell inoculation, when tumours reached a size of ⩾100 mm^3^, PBS liposomes, free ZD6126 (100 mg kg^−1^), PEG-liposomal ZD6126 (100 mg kg^−1^) or RGD-PEG-liposomal ZD6126 (100 mg/kg) was administered i.v. in the tail vein. Tumour size was measured every second day, using digital calipers. For the multiple 100 mg kg^−1^ dose study, mice were also treated for the first time at day 11 followed by two injections at 4-day intervals (days 15 and 19). Tumour volumes were calculated using the following equation: *V*=(*A* × 0.52) × *B*^2^, where *A* is the largest and *B* is the smallest superficial diameter. Mice were killed when tumour reached a volume of 2000 mm^3^, if tumour had broken open through the skin or when animals appeared moribund.

### Animal studies

All animal studies were validated and performed according to Utrecht University ethical guidelines, in line with national laboratory animal regulations.

### Statistical methods

For the necrosis scores, Mann–Whitney rank-sum test was used, and for analysis of the survival curves the Log-rank test was applied.

## Results

### Liposome-encapsulated ZD6126

ZD6126-loaded PEG liposomes or RGD-PEG-liposomes were prepared by the lipid-film hydration method. The amount of encapsulated drug increased proportionally with the total amount of ZD6126 added. A positive correlation between liposome diameter and amount of encapsulated drug was observed, indicating that the ZD6126 is primarily present in the aqueous interior of the liposomes. The liposomes that were used in these studies had a mean diameter of 0.15 *μ*m and a polydispersity index below 0.2, indicating limited variation in particle size. Hydration of 100 mM total lipid with 40 mg ml^−1^ ZD6126 yielded an average concentration of 7.5±2.5 mg ZD6126 per ml of liposome suspension. ZD6126-loaded liposomes showed less than 10% leakage in PBS, at 37 and 4 °C over 3 weeks. Particle size was maintained over this time period.

### *In vitro* cytotoxicity

**Figure 1:**
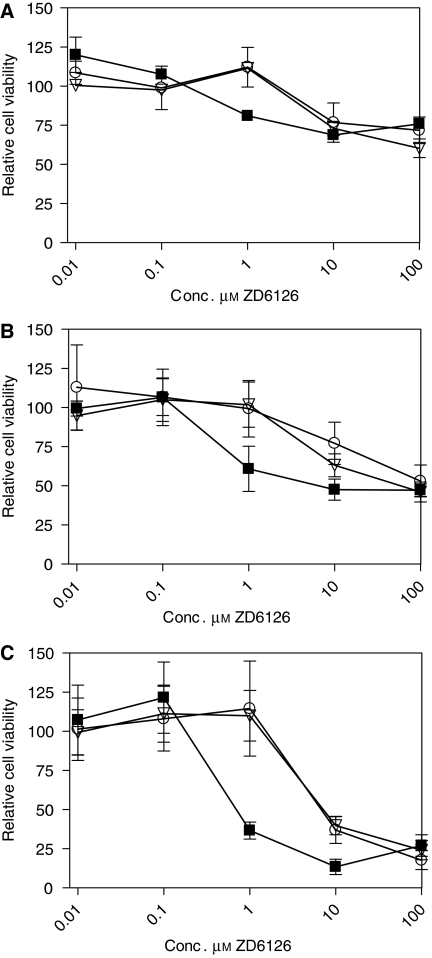
*In vitro* cell toxicity (XTT) after 24 h incubation with free ZD6126 (▪) PEG-liposomal (○) and RGD-PEG-liposomal (▿) ZD6126 formulations. These were tested at a range of concentrations and for following cell lines: human umbilical vein endothelial cells (HUVECs) (**A**) murine melanoma cell line B16.F10 (**B**), and murine tumour-derived macrophages J774A.1 (**C**). Data are calculated against buffer-treated control cells (set to 100% viability).

Cytotoxicity, as measured by XTT assay (Scudiero *et al*, 1988), of free and liposome-encapsulated ZD6126 was examined in three cell types: B16.F10 murine melanoma cells, HUVECs and murine macrophages (J774A.1). After 24 h incubation, ZD6126 at drug concentrations below 1 *μ*M did not induce cytotoxicity (Figure 1A–C). However, ZD6126 concentrations of 1 *μ*M and above for free ZD6126 and 10 *μ*M and above for liposomal ZD6126 reduced viability for all cell types (Figure 1A–C). In contrast, for both RGD-PEG-liposomes and PEG-liposomes, ZD6126 toxicity was detected only at 10 *μ*M and above. These results suggest that over 90% of the drug remains encapsulated in the liposomes for at least 24 h in cell culture medium. Viability of J774A.1 macrophages was reduced by maximum 75% compared with vehicle-treated controls. Human umbilical vein endothelial cells and B16.F10 melanoma cells showed only moderate reductions in cell viability of about 50 and 30%, respectively.

### Pharmacokinetics of ZD6126 and ZD6126-phenol after intravenous administration of liposome-encapsulated ZD6126

**Figure 2:**
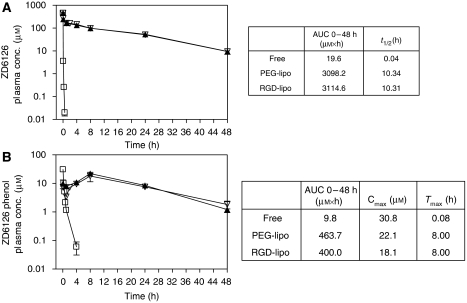
Pharmacokinetics of ZD6126 (**A**) and ZD6126 phenol (**B**) after administration of free and liposomal ZD6126 in non-tumour-bearing C57Bl/6 mice. Formulations were injected in a single bolus intravenous dose of 20 mg kg^−1^. Groups (*n*=3) consisted of free ZD6126 (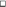), PEG-liposomal ZD6126 (▴), and RGD-PEG-liposomal ZD6126 (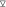). At selected time point, post-injection blood was collected and ZD6126 and ZD6126 plasma levels were measured by tandem mass spectrometry. All points are averages of three mice±s.d. Additionally, AUC values *t*_1/2_, *C*_max_ and *T*_max_ values were calculated.

Intravenously administered free ZD6126 (20 mg kg^−1^) was rapidly (*t*_1/2_=0.04 h) converted to ZD6126-phenol and quickly (*t*_1/2_=0.08 h) cleared from the blood (Figure 2A and B). Both PEG-liposome and RGD-PEG-liposome formulations of ZD6126 showed identical pharmacokinetics. Calculated ZD6126 half-life for both formulations was approximately 10 h, which is typically observed for PEG-liposome-encapsulated drugs in mice (Metselaar *et al*, 2003). ZD6126 concentrations were higher than 10 *μ*M, a level causing *in vitro* cell toxicity, even at 48 h after injection. ZD6126 phenol levels showed increasing plasma levels during the first 8 h after injection. Subsequently, a steady decrease was observed.

### Tissue localisation of RGD-PEG liposomes

**Figure 3:**
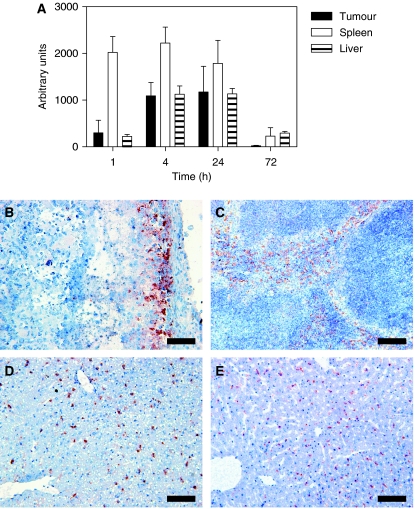
Histological examination of tumour, liver, and spleen. (**A**) Semi-quantification of RGD-stained tumour, spleen, and liver sections over time. Values are calculated per gram tissue. Bars represent means±s.e. (**B**) Tumour rim staining of RGD 24 h after injection of RGD-PEG liposomes containing ZD6126 (20 mg kg^−1^). (**C**) RGD staining is seen only in the red pulp of the spleen (4 h post-injection). (**D**) RGD immunostaining of an RGD-PEG-liposomal ZD6126 (20 mg kg^−1^)-treated mouse liver, excised 1 h after injection. (**E**) Untreated control liver stained for F4/80-positive macrophages (all scale bars represent 100 *μ*m).

To investigate tumour, liver and spleen distribution profile of i.v. injected RGD-PEG liposomes, we performed immunohistochemistry using a rabbit anti-RGD antibody to detect the RGD peptides. Semi-quantification of RGD staining showed high spleen accumulation and comparable tumour and liver accumulation per gram tissue (Figure 3A). Tumour and liver tissue staining for RGD was comparable, although in the tumour RGD, staining was more pronounced in the rim (Figure 3B). In the spleen, a staining pattern largely confined to the red pulp was observed consistent with an association with macrophages (Figure 3C). In liver tissue, RGD staining produced a punctuated macrophage-like staining pattern very similar to that obtained following staining for F4/80 antigen, a marker expressed on a wide range of mature tissue macrophages (Lee *et al*, 1985) (Figure 3D and E).

### Evaluation of tumour necrosis

**Figure 4:**
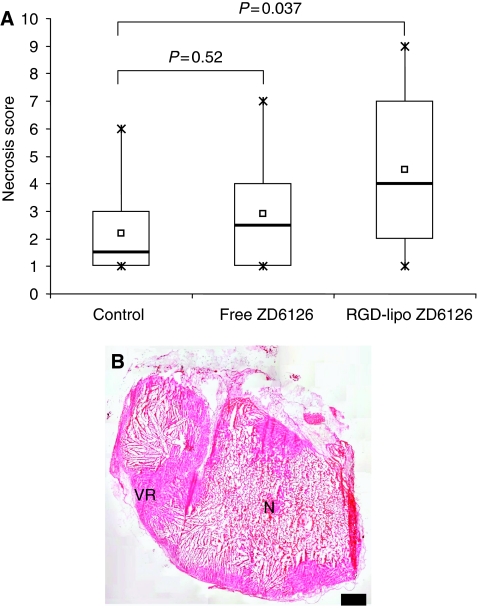
(**A**) Necrotic score of tumour tissue 24 h after administration of vehicle, free ZD6126 100 mg kg^−1^, and RGD-PEG-liposomal ZD6126 100 mg kg^−1^. □, mean of data; —, median; 
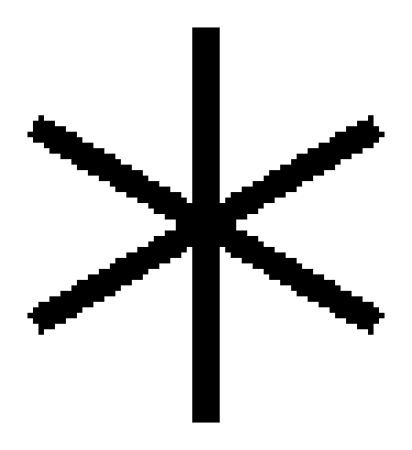
, highest and lowest value; 
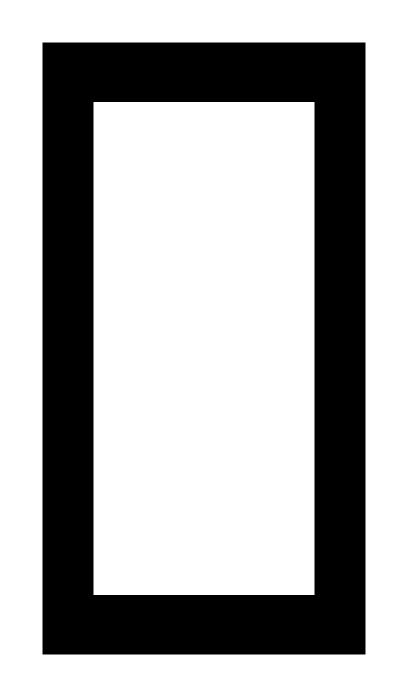
, interquartile range (25–75%). *P*-values: Mann–Whitney rank-sum test. (**B**) Tumour section (H&E-stained) of a mouse treated with 200 mg kg^−1^ free ZD6126, 24 h after intravenous injection. N=necrotic area and VR=viable rim; scale bar represents 1 mm.

B16.F10 melanoma-bearing mice were treated i.v. with 100 mg kg^−1^ free ZD6126, RGD-liposomal ZD6126, or vehicle. Tumours were excised at 24 h after injection. Tumour sections were visually scored for necrosis by a pathologist (FRW) blinded to treatment. Vehicle-treated controls showed some necrosis. In this tumour model, free ZD6126 (100 mg kg^−1^) did not show a significant difference in the degree of tumour necrosis compared with vehicle-treated controls (Figure 4A). In contrast, RGD-liposomal ZD6126 (100 mg kg^−1^ ZD6126)-treated tumours showed a significantly increased tumour necrosis score compared with control. As a control, 200 mg kg^−1^ free ZD6126 was administered and showed >90% necrosis 24 h after injection (Figure 4B).

### Therapeutic efficacy of a single-dose treatment schedule

**Figure 5:**
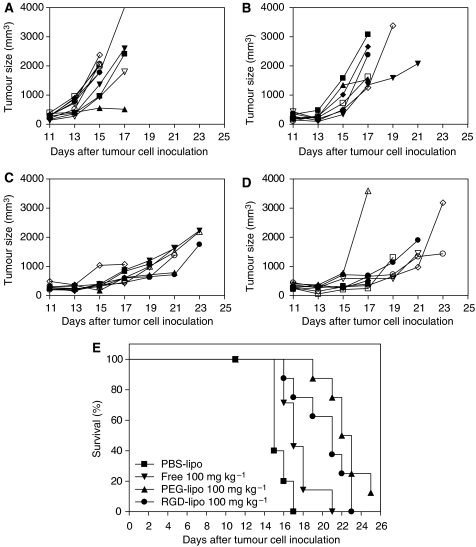
Individual tumour growth curves and survival curve after a single dose of 100 mg kg^−1^ ZD6126 formulations administered when tumours reached ⩾100 mm^3^ (11 days after tumour cell inoculation). (**A**) Control vehicle (*n*=10). (**B**) Free ZD6126 100 mg kg^−1^ (*n*=9). (**C**) PEG-liposomal ZD6126 100 mg kg^−1^ (*n*=8). (**D**) RGD-PEG-liposomal ZD6126 100 mg kg^−1^ (*n*=8). (**E**) Survival curve of all four groups (mice out of experiment when tumours reached >2000 mm^3^, when tumours broke open through the skin and when mice were moribund). *P*-values: vehicle *vs* free ZD6126, *P*=0.0070, vehicle *vs* PEG-liposomal ZD6126, *P*⩽0.0001, vehicle *vs* RGD-PEG-liposomal ZD6126, *P*=0.0004; free ZD6126 *vs* PEG-liposomal ZD6126, *P*=0.0004; free ZD6126 *vs* RGD-PEG-liposomal ZD6126, *P*=0.0315; and PEG-liposomal ZD6126 *vs* RGD-PEG-liposomal ZD6126, *P*=0.0944.

B16F10 melanoma-bearing mice were treated with 100 mg kg^−1^ free ZD6126 or encapsulated in PEG liposomes or RGD-PEG liposomes. Tumour growth was compared with vehicle-treated controls. Growth curves of individual mice showed very rapid growth kinetics for the PEG-liposomal PBS-treated control mice, which are typically observed in this melanoma model (Figure 5A). For mice treated with free ZD6126, an initial growth arrest was seen, which lasted less than 4 days after treatment (Figure 5B). After this initial delay, tumour growth resumed. Both PEG-liposomal (Figure 5C) and RGD-PEG liposomal (Figure 5D) encapsulated ZD6126 arrested tumour growth for much longer periods of time. Statistical comparison of groups was performed using survival analysis. Death of animals was recorded when tumour size exceeded 2000 mm^3^ or when mice appeared moribund. Each of the ZD6126-treated groups showed significant survival advantages over vehicle-treated controls. Between ZD6126 treatments, free drug was less effective than either liposome formulations. Comparing the two liposome formulations, no statistically significant difference was noted.

### Therapeutic efficacy in a multiple-dose treatment schedule

**Figure 6:**
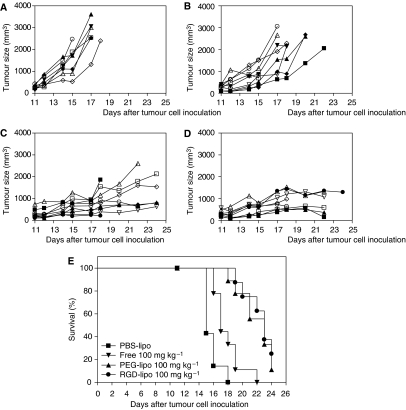
Individual tumour growth curves and survival curve after multiple (3 × every 96 h, days 11, 15, and 19 after tumour cell inoculation) dose of 100 mg kg^−1^ ZD6126 formulations administered when tumours reached ⩾100 mm^3^. (**A**) Control PBS liposomes (*n*=7). (**B**) Free ZD6126 100 mg kg^−1^ (*n*=9). (**C**) PEG-liposomal ZD6126 100 mg kg^−1^ (*n*=9). (**D**) RGD-PEG-liposomal ZD6126 100 mg kg^−1^ (*n*=8). (**E**) Survival curve of all four groups (mice out of experiment when tumours reached >2000 mm^3^, when tumours broke open through the skin or when mice were moribund). *P*-values: vehicle *vs* free ZD6126, *P*=0.0097; vehicle *vs* PEG-liposomal ZD6126, *P*⩽0.0001, vehicle *vs* RGD-PEG liposomal ZD6126, *P*⩽0.0001; free ZD6126 *vs* PEG-liposomal ZD6126, *P*=0.0014; free ZD6126 *vs* RGD-PEG liposomal ZD6126, *P*=0.0006, and PEG-liposomal ZD6126 *vs* RGD-PEG-liposomal ZD6126, *P*=0.5737.

B16F10 melanoma-bearing mice were treated three times (every 4 days) i.v. with 100 mg kg^−1^ free ZD6126 or the same dose encapsulated in PEG liposomes or RGD-PEG liposomes. Tumour growth was compared with vehicle-treated controls (Figure 6A). Similar to the single-treatment regime, growth delay was observed for all three ZD6126 formulations. Free ZD6126 showed a prolonged antitumour effect compared with the single-dose study (Figure 6B). However, tumour size still increased and the majority of animals were killed before the third planned injection on the day after tumour cell inoculation. Also for PEG- and RGD-PEG liposome formulations, a more pronounced tumour growth delay was shown in comparison with the single-dose experiment (Figure 6C and D). However, following the third injection, most animals in the PEG-liposomal ZD6126 and RDG-PEG-liposomal ZD6126 groups became moribund or showed clinical signs within 24 h of injection and were killed.

## Discussion

Vascular disrupting agents have been widely explored as antitumour agents in preclinical and clinical studies. In this study, we have shown substantial improvement in the antitumour activity of the VDA ZD6126 by liposomal encapsulation. Water-soluble ZD6126 was stably associated with the liposomes. *In vitro* cytotoxicity studies confirmed limited leakage from the liposomes. Human umbilical vein endothelial cells, murine melanoma cells (B16.F10), as well as macrophages (J774A.1) all showed a 10-fold decreased cytotoxicity for the liposome-encapsulated ZD6126 as compared with the free drug, which is consistent with <10% drug release in buffer. This conclusion is supported by previous studies that showed limited cellular interaction of PEG liposomes (Du *et al*, 1997; Miller *et al*, 1998) The experiment also showed that ZD6126 cytotoxicity may occur after continuous exposure over 24 h to relatively high drug concentrations, which is in agreement with previous findings (Blakey *et al*, 2002a). For the free drug, such prolonged exposure is unlikely to occur *in vivo* in mice, as both the parent drug ZD6126 and its active moiety ZD6126 phenol have a short half-life. However, as PEG liposomes tend to accumulate in macrophages, sustained high levels of ZD6126 may be reached in this cell type after liposomal administration of ZD6126.

Intravenously administered free ZD6126 and its dephosphorylated active form ZD6126 phenol were cleared from the circulation within 15 min (Figure 2). These observations are in accordance with previously described distribution profiles and pharmacokinetic data for rat and human pharmacokinetics (Blakey *et al*, 2002a; Radema *et al*, 2002; Scurr *et al*, 2004; Beerepoot *et al*, 2006). In contrast, pharmacokinetics of both PEG-liposomal and RGD-PEG-liposomal encapsulated ZD6126 showed a much longer half-life irrespective of the presence of the targeting peptide, which is probably due to macrophage saturation upon high lipid dose administration. A comparison of AUC between free and liposomal ZD6126 translates into a 160-fold increase over a 48 h period. ZD6126 phenol levels after a single dose of liposomal ZD6126 were detectable for at least 2 days. This translates into 40-fold increased AUC values over 48 h period after injection. Levels of ZD6126 phenol in plasma peaked at 8 h after injection. This is probably the result of intracellular processing of the ZD6126-containing liposomes by the MPS. At this time point, the MPS organs are responsible for the majority of liposome clearance from the circulation (Schiffelers *et al*, 2003). Subsequent release of the converted prodrug from the MPS cells into the circulation could explain the observed profile. Quantifying immunohistochemical staining of RGD-PEG liposomes in tumour, liver and spleen tissue revealed high splenic uptake and comparable levels of liver and tumour staining. These findings are consistent with the tissue distribution profiles of RGD-PEG liposomes (Schiffelers *et al*, 2003). The RGD-PEG-liposome staining in the tumour showed some evidence of tumour rim accumulation. The B16.F10 melanoma model used in these studies was not very sensitive to free ZD6126 treatment at the 100 mg kg^−1^ dosing schedule. However, 200 mg kg^−1^ free ZD6126 showed >90% necrosis 24 h after injection (Figure 4B), indicating that this model is intrinsically sensitive. The free drug did not significantly increase the degree of tumour necrosis compared with controls, and the tumour growth delay induced by single or multiple doses of free ZD6126 was modest. In contrast, RGD-PEG-liposomal ZD6126 significantly increased tumour necrosis. This may be the result of an improved tumour localisation of ZD6126 as compared with the free drug leading to increased drug exposure of the endothelial cells in the tumour.

For both RGD-PEG and PEG liposomes, tumour growth delay persisted much longer than with free ZD6126, most likely as a result of increased local and systemic drug exposure. This is in contrast to a single earlier report, using actively targeted RGD-PEG liposomes to deliver the VDA combretastatin A4 (Pattillo *et al*, 2005) where there was no evidence of prolonged tumour growth delay. The unstable association of combretastatin A4 with the liposomal bilayer may be responsible for the absence of substantial antitumour effects. The choice of a water-soluble prodrug such as ZD6126, which is stably entrapped into the aqueous interior, may be critical for successful target tissue delivery.

There was no apparent difference between the two liposome formulations, indicating that the association of RGD-PEG liposomes with tumour endothelium does not improve therapeutic efficacy compared with PEG liposomes, which do not display a specific endothelial cell interaction. To intensify the antitumour effects, a multiple dosing schedule was evaluated. Multiple doses of free drug prolonged the antitumour effects. Also for the liposomal formulations, multiple injections significantly prolonged tumour growth delay; however, after the third injection, some mice became moribund. It is at present unclear why the liposomal ZD6126 was not tolerated after the third injection, as toxicological studies have not been performed, although decreased tolerability could result from an altered tissue distribution profile and/or increased drug exposure. The time interval between doses of liposomal ZD6126 to optimise antitumour effects and minimise toxicity warrants further investigation.

In conclusion, liposomal encapsulation of ZD6126 improves monotherapeutic antitumour activity compared with a similar dose of free ZD6126. The prolonged antitumour effects observed for the liposomal formulations could also be beneficial for combination therapy strategies, extending the time to attack remaining viable tumour cells. RGD-PEG liposomes, targeted to angiogenic endothelial cells, proved to be no more efficacious than passively targeted PEG liposomes, indicating that active targeting and cell-specific delivery of VDAs do not offer an advantage over target tissue delivery in this tumour model. After phosphatase cleavage, ZD6126 phenol can traverse cellular membranes and bind to its intracellular target, tubulin, which could reduce the importance of target cell-specific delivery. This would mean that locally accumulated liposomes serve as a depot for ZD6126, increasing tumour site drug exposure compared with the rapidly cleared free ZD6126. This increase in tumour drug exposure could also induce alternative mechanisms of action for ZD6126, including tumour cell and tumour-associated macrophage toxicity, which could contribute to overall antitumour effects.
